# Providing theoretical insight into the role of symmetry in the photoisomerization mechanism of a non-symmetric dithienylethene photoswitch†

**DOI:** 10.1039/d2cp00550f

**Published:** 2022-05-02

**Authors:** Edison Salazar, Suzanne Reinink, Shirin Faraji

**Affiliations:** Theoretical Chemistry, Zernike Institute for Advanced Materials, University of Groningen Nijenborgh 4 9747 AG Groningen The Netherlands s.s.faraji@rug.nl

## Abstract

Dithienylethene (DTE) molecular photoswitches have shown to be excellent candidates in the design of efficient optoelectronic devices, due to their high photoisomerization quantum yield (QY), for which symmetry is suggested to play a crucial role. Here, we present a theoretical study on the photochemistry of a non-symmetric dithienylethene photoswitch, with a special emphasis on the effect of asymmetric substitution on the photocyclization and photoreversion mechanisms. We used the Spin-Flip Time Dependent Density Functional Theory (SF-TDDFT) method to locate and characterize the main structures (conical intersections and minima) of the ground state and the first two excited states, S_1_ and S_2_, along the ring-opening/closure reaction coordinate of the photocyclization and photoreversion processes, and to identify the important coordinates governing the radiationless decay pathways. Our results suggest that while the main features that characterize the photoisomerization of symmetric DTEs are also present for the photoisomerization of the non-symmetric DTE, the lower energy barrier on S_1_ along the cycloreversion reaction speaks in favor of a more efficient and therefore a higher cycloreversion QY for the non-symmetric DTEs, making them a better candidate for molecular optoelectronic devices than their symmetric counterparts.

## Abstract 2

Dithienylethene photoswitches have shown to be excellent candidates in the design of efficient optoelectronic devices, due to their high photoisomerization quantum yield, for which symmetry is suggested to play a crucial role.

## Graphical abstract

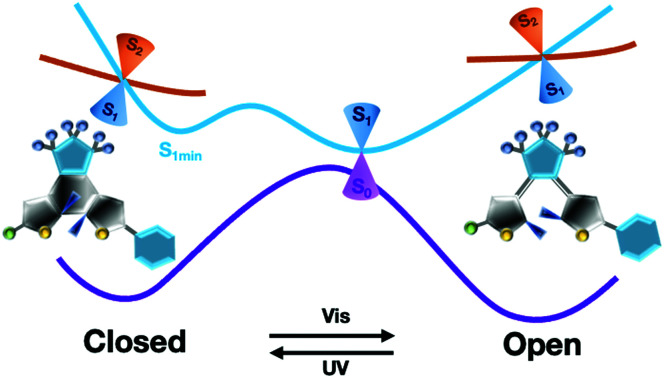

## 1 Introduction

**Fig. 1:**
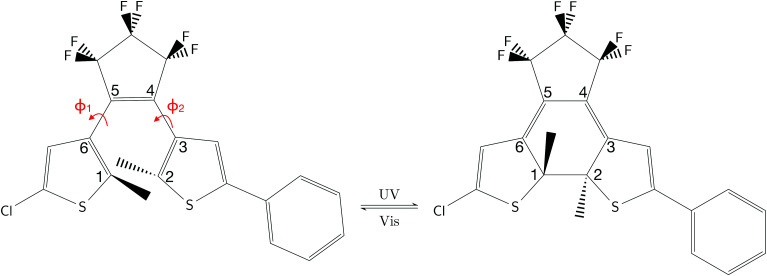
Chemical structure of the DTE studied in this work. Left side represents the open-ring and right side represents the closed-ring. The numbers in the isomers describe the chromophores CHD and cZc-HT, respectively. The torsion angles (in red) *ϕ*_1_ and *ϕ*_2_ are determined by the dihedral angles ∠*C*_1_–*C*_6_–*C*_5_–*C*_4_ and ∠*C*_2_–*C*_3_–*C*_4_–*C*_5_, respectively.

Dithienylethenes (DTEs) are a type of photochromic molecules which can switch between two stable isomers using light. This photoswitching behavior takes place through a cyclization (open-ring (O) to closed-ring (C) isomer) induced by the light absorption in the range of UV and a cycloreversion reaction (closed-ring to open-ring isomer) in the visible range^1,2^ (see Fig. 1). Each isomeric form has different electronic properties, for example, in the open-ring, the discontinuity between the π-systems of the thiophene-rings causes a poor electronic interaction between the thiophene-rings and consequently to the functional groups attached (see Fig. 1). Instead, in the closed-ring, the π-systems of the thiophene-rings are continuous; this creates a delocalization of the π-electrons over the whole molecule^2–5^ making the conductivity between the thiophene-rings easier. Additionally, DTEs show high fatigue resistance,^2,6^ thermal irreversibility,^2,6^ and large quantum yield (QY) of photoisomerization.^2^ These features make them excellent candidates for developing modern technologies, for example, application of molecular photoswitches^1,2^ and photomolecular motors^2,7^ in nanomachines^8^ and molecular electronic devices.^7,9–11^

Several authors have studied numerous DTE derivatives from both experimental^1–4,11–18^ and theoretical^1,2,5,16–25^ perspectives for more than three decades. Although many aspects of DTEs have been clarified, the control of the QY of the photocyclization and photoreversion is still a puzzle. In this respect, it has been observed experimentally that the photoisomerization QY is highly dependent on the substituent groups that are attached to the thiophene-rings.^26^ Furthermore, photocyclization and photoreversion processes of DTEs take place in a small volume space as well as in the range of the picoseconds.^11,27^ Additionally, most DTEs do not show emission,^23^ suggesting the internal conversion process, mediated by conical intersections (CIs), as an active excited-state non-radiative decay channel.^2,17,21^

**Fig. 2:**
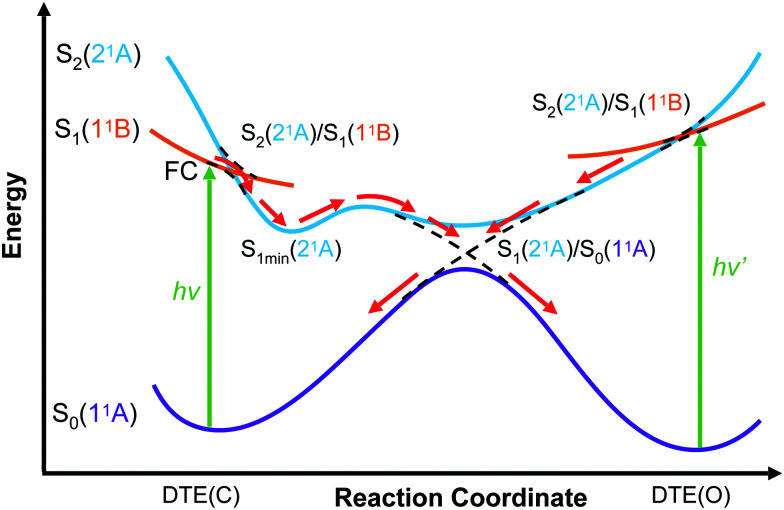
Schematic representation of the photocyclization and the photoreversion reactions of a symmetric DTE. The dashed lines represent the crossing between excited states S_2_(2^1^A) and S_1_(1^1^B), and S_1_(2^1^A) and the ground state S_0_(1^1^A). FC, C and O refer to the Franck-Condon region, closed-ring and open-ring, respectively. Adopted from Fig. 5 of ref. 2.

CIs play an essential role in the excited-state deactivation process of the central backbone of DTEs, the cyclohexadiene (CHD) chromophore.^21^ In fact, the photoisomerization of the CHD triggers the photochromic switching behavior in the DTEs.^28^ It is known that the photochemical interconversion of CHD (isolated) occurs at least through two CIs,^29–34^ and recently we have proposed an alternative deactivation path through a third CI 
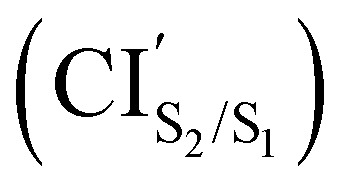
.^34^ Nowadays, it is generally accepted that in DTEs (specifically, symmetric DTEs, *i.e.* DTEs where the attached substituent groups to thiopheny groups are the same), the photocyclization reaction takes place as follow^2^ (see Fig. 2): upon photoexcitation of the open-ring to the bright state S_1_ (this state belongs to the irreducible representation 1^1^B under *C*_2_ symmetry in a symmetric DTE), a rapid decay undergoes on the excited state 1^1^B followed by an internal conversion to the excited state 2^1^A *via* a CI. Afterwards, the DTE moves along 2^1^A, until it reaches the CI_S1(2^1^A)/S0(1^1^A)_. Once this crossing is reached, a decay occurs to either the ground state of the closed-ring or the open-ring. Likewise, the photoreversion process starts after the photoexcitation on the closed-ring has reached the Franck-Condon (FC) region in state S_1_ (this state belongs to the irreducible representation 1^1^B under *C*_2_ symmetry in a symmetric DTE). It is subjected a rapid decay until the DTE undergoes an internal conversion to the excited state 2^1^A *via* a CI. After this CI, the reaction encounters an energy barrier (up to 4.2 kcal mol^−1^ ^2,35–37^ from a local minimum on 2^1^A) in 2^1^A before the DTE reaches the same CI_S1(2^1^A)/S0(1^1^A)_ of the photocyclization reaction. From there, a decay occurs to either the ground state of the open-ring or the closed-ring.

In order to locate and characterise these CIs, theoretical works have been performed on various DTEs, using multireference wavefunction methods,^17,23,38,39^ semi-empirical methods^12,40^ and time dependent density functional theory (TDDFT).^1,41–43^ The complete active space self-consistent field method (CASSCF) is one the most used multireference methods to study photoisomerisation processes of DTEs.^2,28^ Calculations based on these method by Boggio-Pasqua *et al.*^21^ and Asano *et al.*^23^ show the importance of the CIs in the photoisomerization reaction of DTEs during the photoreversion process. Although the results obtained using CASSCF method are consistent with experimental findings (in the region after the local minimum on S_1_(2^1^A)) explaining the correlation between the QY of photoreversion with the substituent groups that are attached to the thiophene-rings,^2,17,23^ it fails to reproduce the correct order of the states S_1_(1^1^B) and S_2_(2^1^A) in the FC region.^17,21,23^ This fact has restricted the use of the CASSCF method to study this mechanism to the states S_1_(2^1^A) and S_0_(2^1^A). This behavior has been reported in previous works focused on the photochemistry of the CHD chromophore.^44–47^ The second-order perturbation theory for the CASSCF method (CASPT2) is also a multireference method widely used to study photoisomerisation processes of DTEs.^2,23,28^ The lack of dynamical correlation in CASSCF that is incorporated in CASPT2 shows a correct order of the S_1_(1^1^B) and S_2_(2^1^A) in the FC region.^23^ It is known that in the photochemical processes, the adequate balance of both dynamical and non-dynamical correlation plays a fundamental role, for example, in the correct description of the energies and CIs.^21,33,40,48^ Recently, Jankowska *et al.*^40^ used a semi-empirical method called orthogonalization- and dispersion-corrected multireference configuration interaction approach (ODM2/MRCI)^49–52^ which allowed to fully include both non-dynamic and dynamic correlation effects. Their results provide for the first time a detailed description of the role of both correlations in the photochemistry of DTEs.

Single reference character methods, such as DFT and TDDFT, have the capability to describe the dynamic correlation energy. Additionally, it has been shown that, for DTEs,^19,24^ TDDFT is able to describe the right ordering for the S_1_(1^1^B) and S_2_(2^1^A) states in the FC region. However, these methods have some important limitations, for example, they can not recover the non-dynamic correlation as well as serious problems to describe CIs^53,54^ (at least CIs between the ground and the excited states), where multi-reference description is a necessary component. An extension to TDDFT proposed by Shao *et al.*,^55^ called spin-flip time-dependent density functional theory (SF-TDDFT), developed to describe diradicals with strong nondynamical correlation, has shown a great performance for describing CIs in ethylene,^56^ uracil,^57^*cis*-stilbene,^58^ CHD^34^ and recently light-driven rotary molecular motors.^59^ Within this approach, a high-spin triplet state is chosen as the initial reference state allowing that the S_0_ and the singlet excited states to be treated at the same footing. SF-TDDFT recovers both nondynamical and dynamical correlation from SF and DFT, respectively.^55,60^ These features have made SF-TDDFT a great alternative to address photochemical processes.

Our recent theoretical study^34^ on the photochemical interconversion between cyclohexadiene and hexatriene has shown that SF-TDDFT can successfully describe and characterize the most important geometries on the potential energy surfaces along the ring-opening/closure reaction coordinate, in an agreement with those obtained by multireference wavefunction methods. Our benchmark calculations on the two fisrt excited states of the CHD revelas that SF-TDDFT, in particular in combination with the BHHLYP functional (see ref. 34 and the Table 1 in the ESI†) shows a reasonable performance compared with wavefunction-based method (XMS-CASPT2^61^ and ADC(2)^62^) and experimental results, suggesting that SF-TDDFT could be a good low-cost method to study complex molecules that contain the CHD chromophore as their central backbone, such as the DTE molecules considered here (Fig. 1).

**Table 1:** Energies (in eV), C_1_–C_2_ bond distances (in Å) and torsion angles (in degrees) for the important geometries of the S_0_, S_1_ and S_2_ PESs obtained with ωB97X-D/cc-pVDZ for the ground state and with SF-BHHLYP/cc-pVDZ for the excited states, in gas phase. The energies are relative to S_0min_ energy of the open-ring. C and O in parenthesis stand for closed-ring and open-ring, respectively and EB stands for the highest energy barrier between the minimum S_1_(C) and CI_S_1_/S_0__

Structures	Energy (eV)	C_1_–C_2_ (Å)	*ϕ*_1_	*ϕ*_2_
S_0_ (C)	0.26	1.54	5.78	6.90
S_1FC_ (C)	3.16	1.54	5.78	6.90
CI_S_2_/S_1__	2.79	1.57	14.91	13.06
S_1min_ (C)	2.58	1.59	8.59	19.95
S_1EB_(C)	2.69	1.75	10.19	23.88
TS_0_	2.16	1.97	23.51	26.53
CI_S_1_/S_0__	2.60	2.08	8.21	24.19
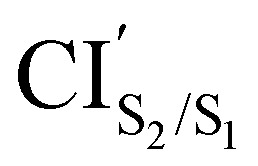	3.70	3.07	29.97	18.78
S_1min_ (O)	3.41	3.22	29.76	40.54
S_2FC_ (O)	4.69	3.50	45.71	48.10
S_0_ (O)	0.00	3.50	45.71	48.10

Controlling the QY of photoisomerization of DTEs has important implications for the design of efficient optoelectronic devices since the efficiency of these devices depend on a large QY (close to 100%) in photocyclization as well as photoreversion.^2,23,38,41^ Although many aspects of the photoisomerization mechanism of the symmetric DTEs have been clarified both experimentally^1,2,13,14,16,17^ and theoretically,^1,2,17,21–23^ to the best of our knowledge, the non-symmetric DTEs remained largely unexplored. It has been shown that non-symmetric DTEs (*i.e.* DTEs with asymmetrically substituted compounds attached to the thiophene-rings) exhibit a similar or in some cases a superior QY of photoisomerization compared to the symmetric counterparts.^2,63,64^ For example, in the extensive review by Irie *et al.*,^2^ the photocyclization and photoreversion QYs of typical DTEs in n-hexane are reported. There, the non-symmetric DTE bearing phenyl/methyl substituent groups shows a similar photocyclization QY with respect to the symmetric counterpart bearing phenyl/phenyl substituent groups. While, it shows a similar photoreversion QY with respect to the symmetric counterpart bearing methyl/methyl substituent groups. Another interesting example is reported in the work by Pariani *et al.*,^64^ where the non-symmetric DTE bearing phenyl/methylthienyl substituent groups shows higher photocyclization and photoreversion QYs with respect to both symmetric counterparts. Furthermore, in the work by Browne *et al.*^15^ on the ring-closure process on a set of asymmetrically substituted DTEs, it is reported that the non-symmetric DTE bearing phenyl/chlorine substituent groups (see Fig. 1) shows a higher percentage (99%) of photostationary states (PSS) for closed-ring with respect to both symmetric counterparts. A high percentage of PSS formed indicates that the QY for photoreversion is considerably lower than the QY for photocyclization. Thus, the primary question of interest that motivates this work is how asymmetry affects the QY of the photoisomerization. Here we provide theoretical insight into the photocyclization as well as photoreversion of the non-symmetric DTE (Fig. 1) and subsequently shed light on its photoizomerization QY, crucial factor for its application as an efficient molecular switch in molecular electronic devices.

This paper is organised as follow: Section 2 describes the computational details. Our results and discussions are presented in Section 3 and finally our concluding remarks are given in Section 4.

## 2 Computational details

Ground state geometry optimization of the open and closed form were performed at the ωB97X-D/cc-pVDZ level of theory,^65,66^ including Grimme's dispersion correction.^67^ Subsequent frequency calculations were undertaken to confirm the nature of the minima. The vertical excitation energies (VEEs) of the first (S_1_) and the second (S_2_) excited state of both isomers and their optimized geometries were obtained using SF-TDDFT(BHHLYP)/cc-pVDZ. Additionally, these excited states were analyzed in terms of natural transition orbitals (NTOs).^68,69^ Furthermore, to take into account the effect of the solvent, the VEEs of S_1_ and S_2_ were computed at SF-TDDFT(BHHLYP)/cc-pVDZ in acetonitrile as solvent, according to the Conductor-like Polarizable Continuum Model (C-PCM) scheme.^70,71^ The dielectric constant (*ε*) and the optical dielectric constant (*ε*_∞_) used to describe acetonitrile were 37.50 and 1.81, respectively. (Un)relaxed potential energy surface (PES) scans of S_0_, S_1_ and S_2_ were carried out, along C_1_–C_2_ bond distance at the SF-TDDFT(BHHLYP)/cc-pVDZ level of theory in gas phase. A state-tracking algorithm, based on the overlap of attachment/detachment densities was used to follow the excited-state character during optimization of the S_1_ and S_2_ excited states. The minimum energy crossing points (MECP) optimisation between S_2_/S_1_ and S_1_/S_0_ were located at the SF-TDDFT(BHHLYP)/cc-pVDZ level of theory by using the penalty function method.^56,58^ All the calculations were performed using Q-Chem 5.3 package.^72^ Cartesian coordinates of all the relevant structures are given in the ESI.†

## 3 Results and discussions

### 3.1 Stationary geometries

**Fig. 3:**
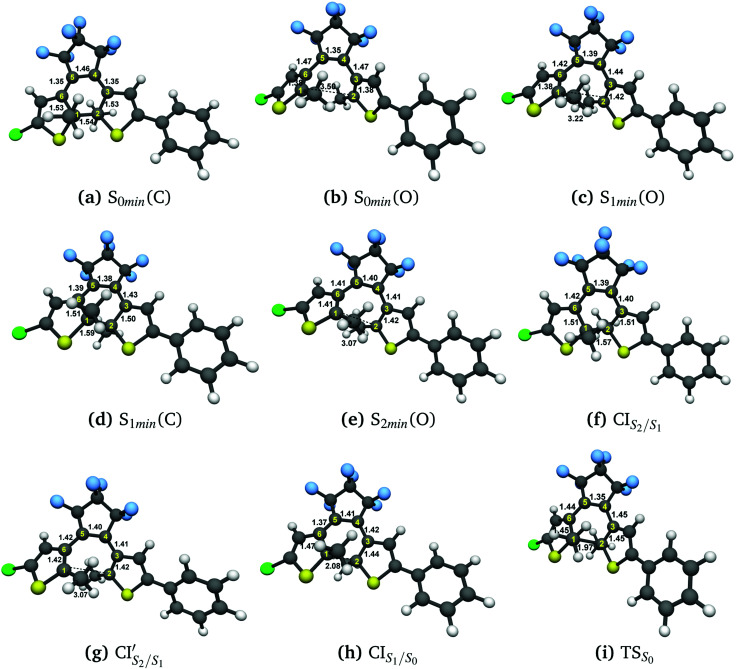
Optimized geometries with DFT(ωB97X-D)/cc-pVDZ: (a) ground state closed DTE, (b) ground state open DTE. Optimized geometries with SF-TDDFT(BHHLYP)/cc-pVDZ: (c) S_1min_ (from Open form), (d) S_1min_ and S_2min_ (from Closed form), (e) S_2min_ (from Open form), (f) CI_S2/S1_ (from Closed form), (g) 
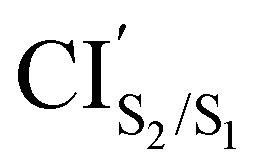
 (from Open form), (h) CI_S1/S0_ (from the highest energy barrier of PES-S_1_) and (i) S_0_ transition state. Distances are in Å.

Fig. 3(a) and (b) illustrate the optimised ground state geometries for the open and closed isomers, respectively. Notice that the C_1_–C_2_ bond/breaking distance is defined as the reaction coordinate. The C_1_–C_2_ distance for the closed-ring is 1.54 Å and 3.44 Å for the open-ring. These distances are very close to those reported in our previous work^34^ carried out on the central backbone of this DTE, the cyclohexadiene (CHD) for the closed-ring and all-*cis*-hexatriene (cZc-HT) for the open-ring. Furthermore, from the geometry point of view, the corresponding central unit in these molecular units have approximately the *C*_2_ symmetry on the S_0min_ (see Fig. 3(a) and (b) and Table 1). In other words, even when CHD and cZC-HT are part of a non-symmetric DTE, these central units trend to conserve their *C*_2_ symmetry in the ground state. In the case of the S_1_ geometry optimisations, the open-ring and closed-ring converged to different minima (see Fig. 3(c) and (d)) with an energy of 2.58 eV, and the C_1_–C_2_ distance of being 1.59 Å for the open-ring, and an energy of 3.41 eV, and the C_1_–C_2_ distance of being 3.22 Å for the closed-ring. Notice that the distance between the reactive carbons C_1_–C_2_ from the S_0min_ does not change (difference 0.05 Å longer) for the closed-ring, but it shortened by 0.28 Å from the S_0min_ for the open-ring. In our previous work, the CHD and cZc-HT molecular units converge to the same minimum which C_1_–C_2_ distance is quite different to the C_1_–C_2_ distance (difference 0.57 Å shorter and 1.06 Å longer for the closed-ring and open-ring, respectively) of the minima found here. However, other works in similar DTEs^2,21,23,38^ show a minimum on the S_1_ state close to the C_1_–C_2_ distance reported here for the S_1min_ (C). A second minimum was located in a C_1_–C_2_ distance similar to the S_0min_ of the open-ring. To the best of our knowledge, a second minimum has not been previously reported in other DTEs. Similarly, the S_2_ state optimisations of the open-ring (see Fig. 3(e)) and closed-ring converged to different minima, with the S_2min_ (C) converging to the S_1min_ (C) (here, we only show the S_1min_ (C); see Fig. 3(d)). The S_2min_ (O) shows an energy of 3.70 eV and the C_1_–C_2_ distance of being 3.07 Å. It must be noted that the distance between the reactive carbons C_1_–C_2_ is shortened by 0.43 Å from the S_0min_ for the open-ring. Interestingly, this structure coincides in geometry and energy with the CI between the S_2_ and S_1_ states. Unlike the S_0min_ for the closed-ring and open-ring, the CHD unit in the S_1min_ (C), and the cZc-HT unit in S_1min_ (O) and S_2min_ (O), do not tend to conserve the *C*_2_ symmetry. It must be noticed that unconstrained geometry optimisation of the S_1_ state from the S_0min_ closed-ring leads directly to a crossing region between the S_2_ and S_1_ states with a difference of 0.03 Å longer than the C_1_–C_2_ distance of the S_0min_ closed-ring. On the other hand, unconstrained geometry optimisation of the S_2_ state from the S_0min_ open-ring leads directly to a crossing region between the S_2_ and S_1_ state with a difference of 0.43 Å longer than the C_1_–C_2_ distance of the S_0min_ open-ring. These crossings will be discussed in more detail later (see Section 3.3). Moreover, our results show an internal conversion between the closed-ring and the open-ring *via* a transition state (see Fig. 3(i)) on the S_0_ state with a C_1_–C_2_ distance of 1.97 Å and an energy barrier of 2.16 eV (49.81 kcal mol^−1^). This energy value indicates that an interconversion between the closed-ring and the open-ring for this DTE can be excluded *via* thermal electrocyclic reaction.

### 3.2 Vertical excitation energies and excited-state characterization

Table 3 summarises the vertical excitation energies (VEEs) for the two lowest singlet excited states of the closed-ring and the open-ring calculated with SF-TDDFT(BHHLYP)/cc-pVDZ in gas phase and acetonitrile as a solvent within the C-PCM scheme, as well as experimental results.^15^ Notice that we have rescrited our study to the singlet excited states since a photocyclization reaction *via* triplet states is present in DTEs bearing chromophores such as transition-metal complexes, perylenebisimide, or triplet sensitizers,^2^ we observe that, both in gas phase and in the acetonitrile solvent, the states with the highest oscillator strength agree with the experimental results; for the closed-ring, the VEE of the brightest state (S_1_) agrees within 0.65 eV and for the open-ring, the VEE of the brightest state (S_2_) agrees within 0.20 eV. In order to characterize these excitations, we used natural transition orbitals NTOs.^68,69^ The hole/electron NTOs provide a compact interpretation of any excited state.^73^ Because we do not observe any significant difference between gas phase and solvent, we restrict our further descriptions just to the brightest states in gas phase (see Fig. S3 in the ESI†).

**Fig. 4:**
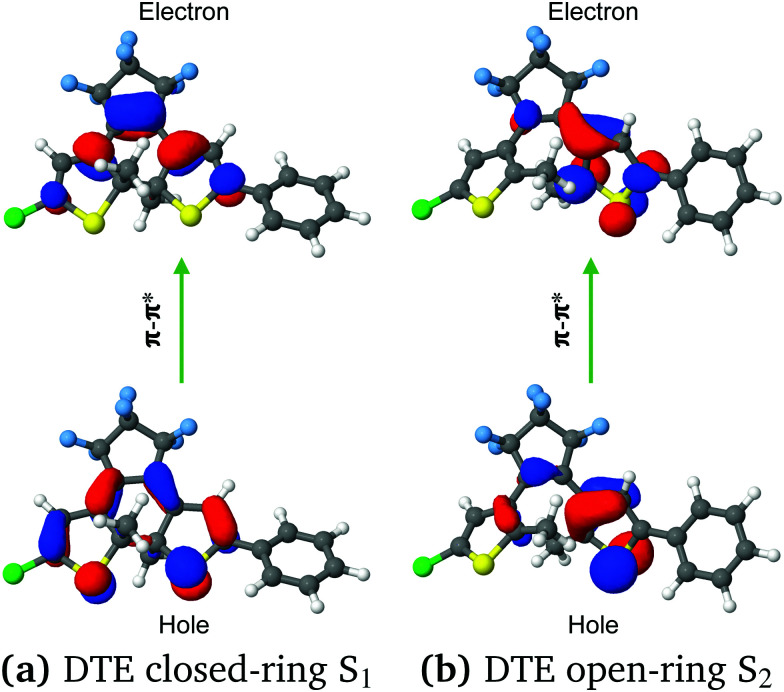
NTOs calculated with SF-TDDFT(BHHLYP)/cc-pVDZ (cutoff value of 0.04) in gas phase for the lowest bright state with significant oscillator strength. (a) DTE closed-ring S_1_, (b) DTE open-ring S_2_.

Fig. 4 depicts the NTOs for the lowest bright state, *i.e.* the S_1_ state for the closed-ring and the S_2_ state for the open-ring, in the gas phase. One can observe that the hole/electron excitations have a π–π* character, and they are distributed mostly over the thiophene-rings and the π-conjugated system of the CHD chromophore for both isomers. In the case of the closed-ring, the hole NTO is localized over the π-conjugated system of the CHD chromophore and the thiophene-rings. Instead, the electron NTO is more localized on the π-conjugated system of the CHD chromophore than the thiophene-rings. Moreover, a close look at the NTOs shape reveals that the interaction between the reactive carbons C_1_ and C_2_ is bonding for the hole NTO and anti-bonding for the electron NTO. This spatial distribution of the hole/electron NTOs and the character of the reactive carbons distance (*i.e.* bonding and anti-bonding) have been observed previously in the frontier orbitals in similar DTEs.^1,24^ In the case of the open-ring, the hole/electron NTOs are localized mostly on the thiophene-ring bearing the phenyl-ring. Here, it is observed a strong contribution of the sulfur atom, for the hole NTO, and the C_2_ atom, for the electron NTO. Unlike the closed-ring, in the open-ring, we can not clearly identify (looking at the shape of the hole/electron NTOs) if the interaction between the reactive carbons C_1_ and C_2_ has a bonding or anti-bonding character. It must be noticed that the shape of the hole/electron NTOs has been observed as well in the frontier orbitals in previous work by Fihey *et al.*^24^ with non-symmetric DTEs. Our previous study on the central unit of this non-symmetric DTE (the CHD and cZc-HT molecular units^34^) has shown that their NTOs are symmetric around these units, for both the closed-ring and the open-ring. The Fig. 4 clearly reveals that the asymmetric substitution leads to an asymmetric distribution of the hole/electron NTOs around the central unit, with the effect being more pronounced for the open-ring. Additionaly, we performed a wavefunction analysis to provide information on the character of the S_0_ → S_1_ and S_0_ → S_2_ transitions. The analysis is based on the squared norm (*Ω*) of the one-particle transition density matrix (1TDM)^74^ between the ground state and the S_1_ and S_2_ excited states, the NTO participation ratio (PR_NTO_) and the number of entangled states (*Z*_HE_).^75^ It must be mentioned that *Ω* is a universal measure of single-excitation character and PR_NTO_ and *Z*_HE_ provide inherent information on the multiconfigurational character of the transition. The value of *Ω* goes typically from 0 to 1, where 1 indicates a pure single excited state. However, values significantly lower than 1 indicate that higher excitations are involved. The PR_NTO_ and *Z*_HE_ descriptors take values close to 1 for states described by a single orbital transition and take higher values when several configurations are involved.^75^Table 2 summarizes the results of the 1TDM and entanglement descriptors. Notice that for the SF-TDDFT method, the wavefunction analysis is performed for each spin (α and β) of the state. Thus, the descriptor *Ω* is defined as the sum of *Ω*_α_ and *Ω*_β_ and for the PR_NTO_ and *Z*_HE_ descriptors the values for each spin α and β have to be analyzed individually. We observe that the total *Ω* of S_1_ for the closed-ring and S_2_ for the open-ring is higher than 0.96 which speaks in favor a single-excitation character. Analyzing the entanglement descriptors, we can see that PR_NTO_ has values close to 1 and *Z*_HE_ has values not higher than 1.5 for all the spins. As expected the values of *Z*_HE_ are somewhat larger than PR_NTO_, this is explained due to the fact that the NTO amplitudes are in general not equal (see ref. 75). Therefore, these values suggest a lower multiconfiguration character for the S_0_ → S_1_ and S_0_ → S_2_ transitions for both closed- and open-ring.

**Table 2:** Wavefunction analysis based on the squared norm (*Ω*) of the one-particle transition density matrix (1TDM), the NTO participation ratio (PR_NTO_) and the number of entangled states (*Z*_HE_)

Descriptors	Closed-ring				Open-ring			
	S_1_		S_2_		S_1_		S_2_	
	α	β	α	β	α	β	α	β
*Ω*	0.411	0.555	0.589	0.227	0.161	0.788	0.769	0.202
PR_NTO_	1.041	1.017	1.010	1.036	1.009	1.002	1.001	1.022
*Z*_HE_	1.500	1.430	1.400	1.430	1.350	1.210	1.230	1.400

**Table 3:** Vertical excitation energies in eV for the two lowest singlet excited states of the open and closed form computed using SF-TDDFT(BHHLYP)/cc-pVDZ in gas phase and acetonitrile solvent. The oscillator strengths are given in parentheses

Environment	Closed-ring/states		Open-ring/states	
	S_1_	S_2_	S_1_	S_2_
Gas phase	2.90(0.574)	3.24(0.017)	4.09(0.037)	4.69(0.161)
Acetonitrile	2.88(0.571)	3.23(0.016)	3.99(0.037)	4.67(0.159)
Experiment^15^ (Acetonitrile)	2.26	—	—	4.86

### 3.3 Minimum energy crossing points

The photoisomerization mechanism of DTEs is essentially controlled by the MECPs between the states^2^: S_2_–S_1_, and S_1_–S_0_. In this DTE, three different MECPs were found, CI_S2/S1_, 
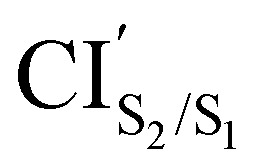
 and CI_S1/S0_. Their relatives energies to S_0min_ of the open-ring are listed in Table 1 and the optimised structures of these geometries are shown in Fig. 3. The calculation of these MECPs along the optimization steps are described in Fig. S1 and S2 (ESI†).

**Fig. 5:**
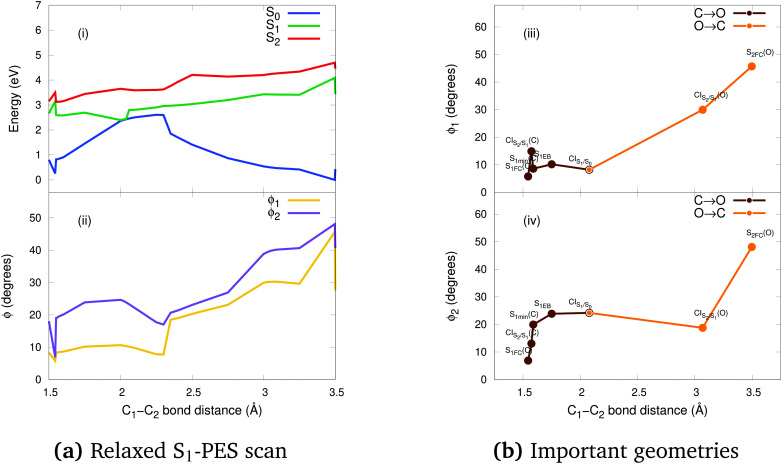
(a) Relaxed S_1_-PES scan: potential energies curves (i) and *ϕ* torsion angles (ii) along the C_1_–C_2_ bond distance obtained with SF-BHHLYP/cc-pVDZ. The energies are relatives to the S_0min_ energy of the open-ring. Notice how the torsion angles change dramatically along the distance of the reactive carbons. (b) Schematic description of the torsion angles *ϕ*_1_ (iii) and *ϕ*_2_ (iv) of the important geometries along the C_1_–C_2_ bond distance of the photocyclization (O → C) and the photoreversion (C → O) processes.

CI_S_2_/S_1__ was obtained starting from closed-ring S_0min_ geometry, with an energy of 2.79 eV and the C_1_–C_2_ distance of being 1.57 Å (see Table 1 and Fig. 3(f)). We can notice that the nature of this structure is rather similar to the closed-ring S_1min_ with respect to the geometry of their CHD molecular units (all the bond distances of their CHD molecular units agree within 0.03 Å), but is energetically 0.21 eV higher. Moreover, the energy of CI_S_2_/S_1__ is 0.37 eV lower than the energy of the closed-ring S_1FC_. Hence, it is natural to assume that the closed-ring S_0min_ will undergo vibrational relaxation downhill from the FC region, towards the CI_S_2_/S_1__, and from there towards the S_1min_. It must be noticed that, this vibrational relaxation is inducing a remarkable twisting motion (around *ϕ*_1_) of the thiophene-ring bearing the chloride group (see Table 1 and Fig. 5(b)-(iii)). Interestingly, a similar twisting motion around *ϕ*_1_ was reported by Asano *et al.*^23^ in a theoretical study on the photochromic cycloreversion reactions of symmetric DTEs.


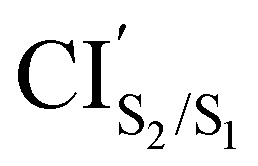
 was obtained starting from the open-ring S_0min_ geometry, with an energy of 3.70 eV and a C_1_–C_2_ distance of being 3.07 Å (see Table 1 and Fig. 3(g)). We can notice that the nature of this structure is rather similar to the open-ring S_2min_. In fact, we do not observe any significant difference between their geometries, symmetries with respect to their cZc-HT chromophores (*C*_2_ symmetry is almost retained in both cZc-HT chromophores) and energies. The latter speaks in favour of the fact that both structures are located extremely close to each other. Notice that the energy of the 
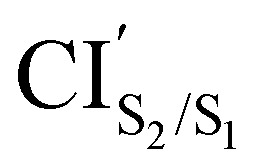
 is 0.99 eV lower than the energy of the open-ring S_2FC_. Thus, it is natural to assume that the open-ring S_0min_ will undergo vibrational relaxation downhill from the FC region and reach the 
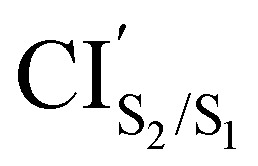
 and from there towards the S_2min_.

CI_S1/S0_ was computed starting from a geometry related to highest energy point on a relaxed PES scan on S_1_ state. All attempts to optimize this crossing did not converge within 500 steps using the penalty function method. The reason is that after the first 40th optimization steps, the states show a high spin contamination, *i.e.* 〈*Ŝ*^2^〉≈ 1.5. It must be noted that states with 〈*Ŝ*^2^〉< 1.2 are assigned as singlet states. This high spin contamination causes an oscillation of the energies of the excited states S_1_ and S_0_ along the optimization steps^76,77^ (see Fig. S2(i) in the ESI†). One way to avoid such an issue is to take a look at the structures along the S_1_/S_0_ MECP optimization steps with high non-adiabatic coupling norm values (see Fig. S2(ii) in the ESI†) and analyze the orbitals involved at these structures in order to select the closest one to the CI_S1/S0_. Another way to tackle this issue is using the spin-adapted spin-flip DFT method (SA-SF-DFT).^76,77^ This method is free of spin contamination, thus one can get proper singlet excited states. Because this method lacks analytical gradients, it can be used as a spot-check along the optimization steps in the region of high spin contamination. Using the former protocol from a set of structures with high nonadiabatic coupling (NAC) norm values, we chose the CI_S1/S0_ structure (see Table 1 and Fig. 3(h)) with the lowest difference of energy between S_0_ and S_1_ computed with the SF-TDDFT method (<0.00014 eV) and SA-SF-DFT method (<0.2 eV) and the highest NAC norm value (745). Interestingly, the geometry of the CHD molecular unit of this CI shows close similarities (0.03 Å lower for C_1_–C_2_, 0.02 Å lower for C_6_–C_1_, 0.01 Å lower for C_4_–C_5_ and C_5_–C_6_, and 0.00 Å for C_2_–C_3_ and C_3_–C_4_) with the corresponding geometry of the CHD molecular unit of the CI_S1/S0_ point obtained by Boggio-Pasqua *et al.*^21^ in an *ab initio* molecular orbital study of three different diarylethene derivatives using the CASSCF method. The energy of the CI_S1/S0_ is 0.19 eV and 1.10 eV lower than the energies of the CI_S2/S1_ and 
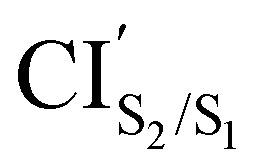
, respectively. Therefore, it is natural to assume that, both the open-ring and the closed-ring will go downhill from the CI_S2/S1_ and 
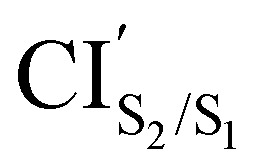
, in the corresponding relaxation processes, towards the CI_S1/S0_, and from there, towards either the open-ring S_0min_ or the closed-ring S_0min_. In fact, comparing the energies (difference of 0.44 eV) and the geometries (difference of 0.11 Å for the bond distance C_1_–C_2_) between the CI_S1/S0_ and the TS_0_, both structures are quite similar.

### 3.4 Potential energy surface

**Fig. 6:**
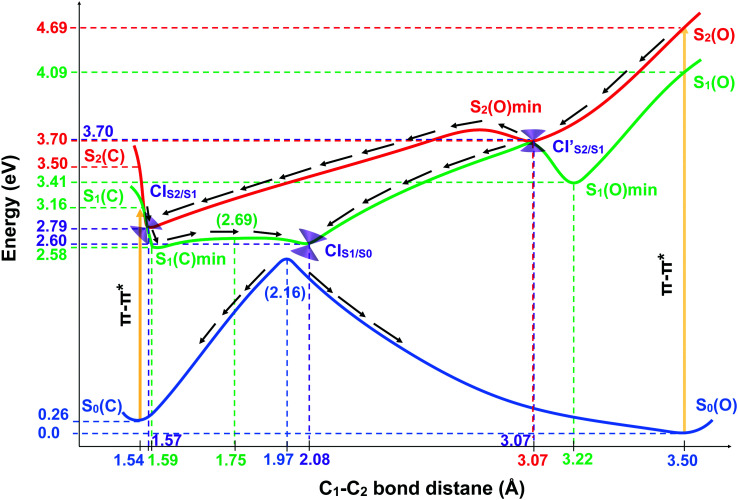
Schematic representation of the PESs of DTE Open form/Closed form photochemical interconversion. The reaction coordinate is the C_1_–C_2_ bond distance. The ground state is in blue, the state S_1_ is in green and the state S_2_ is in red. The curves are a pictorial description that connects the important geometries computed with SF-BHHLYP/cc-pVDZ. The ground state curve is a PES scan trough the C_1_–C_2_ bond distance computed with SF-BHHLYP/cc-pVDZ. The cones in purple represent the MEPCs. The arrows depict how the photochemical interconversion process follow after an absorption of a photon generates the population of the second excited state (S_2_) of open form and the first excited state (S_1_) of closed form. The energies are relatives to the S_0min_ energy of the open-ring.

In order to shed light on the photocyclization and photoreversion processes of the non-symmetric DTE considered in this work, we analyzed the main features of the lowest three singlet states (S_0_, S_1_ and S_2_) computed by (un)relaxed PES scans along C_1_–C_2_ coordinate with SF-BHHLYP/cc-pVDZ. Here, we only show the relaxed PES scan on S_1_ in Fig. 5. The analogous PES scans are represented in the ESI.† An outline of this analysis is depicted in Fig. 6. As it is shown in Fig. 6, the S_0_ PES has two minima corresponding to the closed-ring with C_1_–C_2_ distance of 1.54 Å and the open-ring with C_1_–C_2_ distance of 3.50 Å. Connecting these minima, we found a transition state structure at 1.97 Å along C_1_–C_2_ coordinate with an energy barrier of 2.16 eV (49.81 kcal mol^−1^). This large energy barrier shows that an operative reaction path for an interconversion between the closed-ring and the open-ring for this DTE can be *via* photoexcitation and excludes a thermal electrocyclic reaction as a likely mechanism.

#### Ring-closing reaction

Upon light absorption, the ground state of the open-ring isomer is vertically excited to the bright state S_2_ with an energy of 4.69 eV. From the FC region, the open-ring undergoes a vibrational relaxation which induces a C_1_–C_2_ bond shortening (0.43 Å shorter) as well as smaller torsion angles (*ϕ*_1_ and *ϕ*_2_ being 15.74 and 29.32 degrees smaller, respectively), and subsequently hits the 
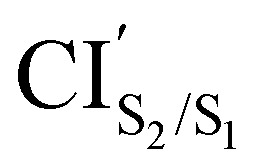
 where occurs an internal conversion. This behavior is typical of processes when the CI is accessible from the FC region without significant energy barriers, as is the case here. From this point, the reaction pathway may bifurcate in two branches, depending on which adiabatic state the wavefunction takes after the system leaves the crossing region. Hence, we can define two scenarios; (1) if the adiabatic S_2_ state changes to S_1_, then the system will evolve directly towards the CI_S_1_/S_0__ (at 2.08 Å with an energy of 2.60 eV) that triggers an ultra-fast internal conversion process and provides a funnel of fast access to the ground state, on which the system can evolve either to the S_0min_ of closed-ring or the S_0min_ of open-ring. Notice that this relaxation path is rather similar to the path of the symmetric DTEs described by Irie *et al.*^2^ in their review about photochromism of diarylethene molecules. (2) if the adiabatic S_2_ state does not change, then the system evolves towards the CI_S_2_/S_1__, and from there, towards the S_1min_ (C) (at 1.59 Å and energy of 2.58 eV). There, the excess of vibrational energy after photoexcitation should enable the system to overcome the energy barrier (0.11 eV higher) and to hit again the CI_S_1_/S_0__, that acts as a doorway for an ultra-fast internal conversion to the ground state, either to the open-ring S_0min_ or the closed-ring S_0min_.

#### Ring-opening reaction

Upon light absorption, the ground state of the closed-ring isomer is vertically excited to the bright state S_1_ with an energy of 3.16 eV. From the FC region, the closed-ring undergoes a vibrational relaxation which induces a C_1_–C_2_ bond elongation (0.03 Å larger) as well as larger torsion angles (*ϕ*_1_ and *ϕ*_2_ being 9.13 and 6.16 degrees larger, respectively), and subsequently hits the CI_S_2_/S_1__ where occurs an internal conversion. From there, the system evolves towards the S_1min_(C), where the excess of vibrational energy after photoexcitation should enable the system to overcome the energy barrier (2.5 kcal mol^−1^ higher) and to hit the CI_S_1_/S_0__. Notice that the nature of this energy barrier as TS on S_1_ has been confirmed using the intrinsic reaction coordinate (IRC) algorithm, which is essentially a series of steepest descent steps going downhill from the TS to the adjacent minimum which is S_1min_(C). At the CI, an ultra-fast internal conversion takes place to the ground state, either to the open-ring S_0min_ or the closed-ring S_0min_. It must be noted that this relaxation path is rather similar to the path of the symmetric DTEs described by Irie *et al.*^2^ in their review about photochromism of diarylethene molecules.

In general, we observe that upon photoexcitation and the subsequent vibration relaxation, the torsion angles *ϕ*_1_ and *ϕ*_2_ as well as the C_1_–C_2_ bond distance undergo significant changes for both ring-opening and closing reactions, making them crucial coordinates in the radiationless pathways. For example, in the case of the photoreversion process, the twisting motion around *ϕ*_1_ governs the pathways connecting the FC region at S_1_ with the S_1min_(C) through the CI_S_2_/S_1__ (see Fig. 5(b)-(iii)). A similar twisting motion around *ϕ*_1_ was also reported by Asano *et al.*,^23^ in their work focused on the photoreversion reaction of symmetric DTEs. In summary, we observe that the principal features that characterize the photoreversion and photocyclization processes in symmetric DTEs^16,17^ are also present for the photoisomerization of this non-symmetric DTE, for example, the energy barrier (S_1EB_(C)) on S_1_ in the cycloreversion process and no energy barrier in the cyclization process, as well as the presence of the CI_S_1_/S_0__ for both electrocyclic reactions. However, it must be noted that the energy barrier (S_1EB_(C)) on S_1_ along the cycloreversion process for the non-symmetric DTE considered in this work is around 2.5 kcal mol^−1^ that is almost half of the enery barrier predicted experimentally^2,35–37^ (around 4.2 kcal mol^−1^) for the symmetric DTEs, which can potentially lead to a higher cycloreversion QY for the non-symmetric DETs compared to the symmetric counterparts. The latter should be further investigated since in a recent study,^36^ on the dynamics of the cycloreversion reaction of a photochromic DTE derivative, it is shown that the QY of the photoreversion process depends on the branching ratio around the CI_S_1_/S_0__ rather than the energy barrier on S_1_.

Furthermore, in previous theoretical works,^17,38^ it has been shown that the energy difference between the CI_S_1_/S_0__ and S_1min_(C) is correlated with the experimental QYs of the photoreversion process. Although these works were performed using the CASSCF method, which lacks the dynamic correlation, the energy difference between the CI_S_1_/S_0__ and S_1min_ (C) may be used as an index of the cycloreversion QY.^38^ Thus, when the energy difference between the CI_S_1_/S_0__ and S_1min_(C) is positive, the efficiency to reach the CI decreases^2^. For the non-symetric DTE considered here, the computed energy difference between the CI_S_1_/S_0__ and S_1min_ (C) is 0.46 kcal mol^−1^ (0.02 eV, see Table 1). To the best of our knowledge, the QYs of the photoreversion and photocyclization processes are not reported for the non-symmetric DTE considered in this work. However, in the work by Browne *et al.*,^15^ it has been concluded that the QY of the ring-opening is considerably lower than the QY of the ring-closing for this non-symmetric DTE which is in agreement with our analysis and results presented here.

In summary, comparing the calculated PESs along ring-opening and closing reactions and the corresponding structures for this non-symmetric DTE with the experimental and theoretical results^17,38^ for the symmetric DTEs, we observe that while there exists a close resemblance between the photoreversion of this non-symmetric DTE with the symmetric counterparts,^78,79^ the lower energy barrier (S_1EB_(C)) on S_1_ along the cycloreversion reaction speaks in favor of a more efficient and higher QY of the cycloreversion process for the non-symmetric DTEs. Additionally, the computed energy difference between the CI_S_1_/S_0__ and S_1min_(C) suggests that the QY photoreversion for this non-symmetric DTE resemble more the symmetric counterpart with Chlorine atom which shows a higher photoreversion QY than the symmetric counterpart with the phenyl substituents.^78,79^

## 4 Conclusions

In the present study, we have used the SF-TDDFT(BHHLYP)/cc-pVDZ level of theory to describe and characterize the photochemistry of a non-symmetric DTE. Our results show how the geometric changes induced by the vibration relaxation after the initial photoexcitation play an important role in the deactivation processes, mainly, the torsion angle *ϕ*_1_ for the photoreversion reaction. Moreover, while the main features that characterize the photoisomerization of a symmetric DTE are also present for the photoisomerization of this non-symmetric DTE, the lower energy barrier (S_1EB_(C)) on S_1_ along the cycloreversion reaction speaks in favor of a more efficient and higher QY of the cycloreversion process for the non-symmetric DTEs. Additionally, the computed energy difference between the CI_S_1_/S_0__ and S_1min_ (C) suggests that the QY photoreversion for this non-symmetric DTE resemble more the symmetric counterpart with Chlorine atom which shows a higher photoreversion QY than the symmetric counterpart with the phenyl substituents. Finally, the SF-TDDFT method shows excellent performance in reproducing important geometries as well as features reported in previous works on DTEs where multireference methods were used. This suggests that SF-TDDFT could be a cheaper alternative to the multireference methods to study complex systems with the same degree of complexity and size as the non-symmetric DTE considered in this work, that are relevant for molecular electronic devices.

## Conflicts of interest

There are no conflicts to declare.

## Supplementary Material

CP-024-D2CP00550F-s001
