# Supplementary material for: Providing theoretical insight into the role of symmetry in the photoisomerization mechanism of a non-symmetric dithienylethene photoswitch
Source: Phys Chem Chem Phys. 2022 May 2;24(19):11592–602. doi: 10.1039/d2cp00550f (PMC9116444; doi:10.1039/d2cp00550f)
Supplement: CP-024-D2CP00550F-s001 [file CP-024-D2CP00550F-s001.pdf]

Electronic Supplementary Material (ESI) for Physical Chemistry Chemical Physics.
This journal is © the Owner Societies 2022

ELECTRONIC SUPPLEMENTARY INFORMATION

Providing theoretical insight into the role of symmetry in the photoisomerization mechanism of a non-symmetric dithienylethene photoswitch

Edison Salazar[a] and Suzanne Reinink[a] and Shirin Faraji[a*]

[a]Theoretical Chemistry, Zernike Institute for Advanced Materials, University of Groningen, Nijenborgh 4, 9747 AG Groningen, The Netherlands

ARTICLE HISTORY
Compiled May 2, 2022

Optimized important geometries

The Cartesian geometry of critical points located on S_0, S_1 and S_2, and their nuclear repulsion energy are given below.

`Closed-form, S0 optimized geometry. E_NN= 3672.3273952721 hartrees`

1	C	-1.9426968527	0.7739896311	-0.5676212039
2	C	-0.7286599020	1.1930192979	-0.1541059399
3	S	-2.1004724089	-0.9318748547	-0.9788487037
4	C	-0.4080368434	-1.2599707797	-0.2742974746
5	C	0.2393270962	0.1258141043	-0.1476845937
6	C	1.5811208151	0.1663584945	-0.0085448696
7	C	2.4721040166	1.3245814749	0.3128446033
8	C	3.6646396227	0.6453981881	1.0413827374
9	C	3.7878156069	-0.7480157990	0.3604907498
10	C	2.3936678970	-1.0370919619	-0.1033768826
11	C	1.8860472638	-2.1926004737	-0.5882486315
12	C	0.4760839974	-2.1412614539	-1.1821545907
13	S	-0.0639027212	-3.9144001523	-1.2618696393
14	C	1.5901454742	-4.4839571722	-0.9862545926
15	C	2.4688712143	-3.5011961919	-0.6603696417
16	Cl	-3.3329815999	1.7774673524	-0.7324244159
17	C	-0.5866153562	-1.8228466595	1.1500086791
18	F	1.8982474617	2.2791659528	1.0776144182
19	F	2.9370008629	1.9357256762	-0.8099760166
20	F	3.3265676295	0.4674632723	2.3366981280
21	F	4.7943032953	1.3594079819	0.9838926086
22	F	4.2849667590	-1.6721315646	1.2173487586
23	F	4.6664006947	-0.6630656934	-0.6732064551
24	H	-0.4958512324	2.2246523341	0.0991039090

CONTACT Shirin Faraji. Email: s.s.faraji@rug.nl

25	C	0.5981253719	-1.6151805973	-2.6266433962
26	C	1.8901992093	-5.9194528356	-1.1073479856
27	H	3.5065332518	-3.6928111113	-0.3929529097
28	C	0.8995068031	-6.8864687108	-0.8858953000
29	C	3.1859739244	-6.3421779355	-1.4417059797
30	C	3.4833394991	-7.6968858031	-1.5392592179
31	C	2.4923048506	-8.6512037044	-1.3087299814
32	C	1.2005140212	-8.2417327858	-0.9829315930
33	H	-0.1127354397	-6.5765101599	-0.6175887085
34	H	3.9598749162	-5.6012659789	-1.6476704743
35	H	4.4940303596	-8.0092443899	-1.8066601609
36	H	0.4209062158	-8.9828044461	-0.8002366480
37	H	2.7260454913	-9.7141053723	-1.3889790732
38	H	-1.1730051622	-1.1142237826	1.7503349239
39	H	0.3940305888	-1.9578626618	1.6297181278
40	H	-1.1074114305	-2.7883384688	1.1325796885
41	H	0.9987601365	-0.5907026693	-2.6262892300
42	H	-0.3747459098	-1.6145113407	-3.1339088329
43	H	1.2886405120	-2.2590782497	-3.1877341896

Open-form, S0 optimized geometry. E_NN= 3597.4015904836 hartrees

1	C	-1.8061537969	1.3952730060	1.6150853719
2	C	-0.5484355257	1.4949956968	1.1126999293
3	S	-2.0740121846	-0.1177009150	2.4239270191
4	C	-0.4579687782	-0.6502704508	2.0728254669
5	C	0.2253308966	0.3090082498	1.3614083575
6	C	1.6048914381	0.1718895080	0.8862924584
7	C	2.5656196908	1.3250056665	1.0174109303
8	C	3.9567316819	0.6824206858	0.8139082419
9	C	3.6447446296	-0.5823249972	-0.0204871471
10	C	2.1927273924	-0.8730564083	0.2644885047
11	C	1.5746220947	-2.1156585933	-0.2098080875
12	C	0.4198084519	-2.1542615629	-0.9595375014
13	S	-0.0253876910	-3.7942002750	-1.2963388977
14	C	1.3385106438	-4.4371215896	-0.4210250533
15	C	2.0996972670	-3.4179259412	0.0781972493
16	Cl	-3.0627286594	2.5726245876	1.5150339801
17	C	0.0055686281	-1.9902193985	2.5507599708
18	F	2.4750711161	1.9626929389	2.2001896308
19	F	2.3439193312	2.2560548976	0.0454078660
20	F	4.4525927261	0.3174448964	2.0126452664
21	F	4.8310902608	1.5011675364	0.2163786423
22	F	4.4665055353	-1.6043829825	0.3216010461
23	F	3.8459440303	-0.3370437439	-1.3368404561
24	H	-0.1769447605	2.3656691000	0.5768405718
25	H	-0.4653809552	-2.2586393439	3.5066180757
26	H	-0.2271193386	-2.7780385546	1.8170322524
27	H	1.0951510399	-1.9841805910	2.6938210542
28	C	-0.4098136680	-1.0222585554	-1.4781389951

29	H	-0.8117632365	-1.2466441222	-2.4760853337
30	H	0.1970914835	-0.1090930921	-1.5480388394
31	H	-1.2564991006	-0.8080114371	-0.8062253085
32	C	1.5609673206	-5.8872387443	-0.2938855573
33	H	2.9930974166	-3.5800513036	0.6777561269
34	C	0.4871562649	-6.7858193900	-0.2274041377
35	C	2.8672257813	-6.3921923310	-0.2193636705
36	C	3.0902155070	-7.7575672730	-0.0684791500
37	C	2.0150265603	-8.6432573172	-0.0029206930
38	C	0.7133265339	-8.1522601093	-0.0872362046
39	H	-0.5373604438	-6.4092121849	-0.2688350886
40	H	3.7141070797	-5.7084500035	-0.2996899503
41	H	4.1130335990	-8.1342434807	-0.0130033080
42	H	-0.1354392048	-8.8361502152	-0.0346083301
43	H	2.1919929425	-9.7139718628	0.1116436975

Closed-Form S1min, S1 optimized geometry. E_NN= 3692.96514181 hartrees

1	C	-1.9483224881	0.6790960369	-0.4466878857
2	C	-0.7041686418	1.0664523625	-0.0571493635
3	S	-2.1157695740	-1.0469605954	-0.7250509106
4	C	-0.3839110490	-1.3632258583	-0.1636545673
5	C	0.2123522279	0.0068100611	0.0478229819
6	C	1.5982094568	0.0815968972	0.0960398638
7	C	2.4234923850	1.2938506268	0.3734191429
8	C	3.7968857070	0.7067551545	0.7530156039
9	C	3.8566759696	-0.6303334357	-0.0127071851
10	C	2.4460514057	-0.9696251731	-0.2034181664
11	C	1.8941672149	-2.2031534188	-0.6792550563
12	C	0.5085739984	-2.1057873869	-1.2492624342
13	S	0.0154981490	-3.8340088073	-1.5830208325
14	C	1.5285393534	-4.4847699220	-1.0768906915
15	C	2.4152975289	-3.4550226334	-0.6365457341
16	Cl	-3.2940219326	1.7132212329	-0.7014182402
17	C	-0.4262081661	-2.1554937508	1.1437665896
18	F	1.9280116606	2.0906756435	1.3304692458
19	F	2.5478123330	2.0455375355	-0.7411213617
20	F	3.8145985435	0.4695582976	2.0724511958
21	F	4.8034992775	1.5274601869	0.4604791750
22	F	4.5623688036	-1.5653930104	0.6770666669
23	F	4.5482353419	-0.4609982514	-1.1705613189
24	H	-0.4331526627	2.1011687091	0.1064228836
25	C	0.5295145960	-1.3393831400	-2.5751758925
26	C	1.8101500859	-5.8858252194	-1.0861247809
27	H	3.4034766564	-3.6522074234	-0.2493366456
28	C	0.8266828607	-6.8418553243	-1.4158872254
29	C	3.1022332349	-6.3514451203	-0.7640392776
30	C	3.3894730969	-7.7016276914	-0.7767391919
31	C	2.4056651555	-8.6290482141	-1.1027769968
32	C	1.1228322859	-8.1880127524	-1.4211109740

33	H	-0.1784373343	-6.5202872396	-1.6625736898
34	H	3.8838184522	-5.6479424201	-0.5128303516
35	H	4.3902794888	-8.0358339025	-0.5297809042
36	H	0.3501604505	-8.9044804502	-1.6739907919
37	H	2.6348062967	-9.6879543829	-1.1087752974
38	H	-0.9798027315	-1.5856678781	1.8917570336
39	H	0.5931361016	-2.3126567599	1.5073303222
40	H	-0.9112390794	-3.1248253908	1.0023316018
41	H	0.8633420275	-0.3175438607	-2.3845998155
42	H	-0.4715488112	-1.3135038313	-3.0097944959
43	H	1.2197223245	-1.8172394998	-3.2735922280

Open-Form S1min, S1 optimized geometry. E_NN= 3625.42070498 hartrees

1	C	-1.7466584825	1.3581221430	1.7060063168
2	C	-0.4860551217	1.4998237707	1.2569648521
3	S	-2.0437028152	-0.2166314295	2.3831930113
4	C	-0.4071350729	-0.7106994602	2.0488731866
5	C	0.2961229198	0.3002315069	1.4170169717
6	C	1.6073876419	0.1950650768	0.8712103856
7	C	2.4971018903	1.3650228045	0.6872357947
8	C	3.9009150217	0.7439893319	0.6021073689
9	C	3.6488162534	-0.6583554843	0.0197849707
10	C	2.2355768619	-0.9242693745	0.3344596930
11	C	1.5962732872	-2.1501959776	-0.0562684971
12	C	0.3437117347	-2.1706891958	-0.7269598107
13	S	-0.2176380481	-3.7801497257	-0.9991145025
14	C	1.2498717496	-4.4715283620	-0.3210533646
15	C	2.1016926456	-3.4231414274	0.1402247334
16	Cl	-3.0061136449	2.5319497269	1.6374209626
17	C	0.0963796838	-2.0093425601	2.5865020258
18	F	2.4222943545	2.2962546856	1.6515947805
19	F	2.2330988560	2.0208713984	-0.4828861176
20	F	4.4003832091	0.6333756057	1.8413540380
21	F	4.7505443881	1.4652954503	-0.1284496144
22	F	4.5150113104	-1.5718231361	0.5409971419
23	F	3.9093591450	-0.6576078685	-1.3125983353
24	H	-0.1064164903	2.3983374787	0.7913617535
25	H	-0.2714572697	-2.1728453415	3.6050692921
26	H	-0.2037898373	-2.8753917947	1.9849868390
27	H	1.1876478480	-1.9869164145	2.6168664090
28	C	-0.4105998982	-1.0139935765	-1.2611527287
29	H	-0.8419303588	-1.2522399880	-2.2377557291
30	H	0.2567253016	-0.1556805039	-1.3445858413
31	H	-1.2249171593	-0.7372996936	-0.5815610558
32	C	1.4890385265	-5.8573659858	-0.2625348756
33	H	3.0494830721	-3.5975312586	0.6245211328
34	C	0.5272809983	-6.8083702782	-0.6982666893
35	C	2.7257064992	-6.3469487501	0.2404376416
36	C	2.9736705287	-7.6994964510	0.2979365999

37	C	2.0136648270	-8.6150656707	-0.1350769671
38	C	0.7905705032	-8.1554562604	-0.6335775289
39	H	-0.4278542972	-6.4706728538	-1.0829358195
40	H	3.4818185107	-5.6503068125	0.5750338289
41	H	3.9230587648	-8.0526505960	0.6825279763
42	H	0.0435294525	-8.8644646776	-0.9701849262
43	H	2.2142927105	-9.6784080706	-0.0847053028

Open-Form S2min, S2 optimized geometry. E_NN= 3624.30231202 hartrees

1	C	-1.6553627321	1.5846255255	1.6596153824
2	C	-0.3823433171	1.6468874642	1.2233021978
3	S	-2.0777989512	0.0058612929	2.2941978935
4	C	-0.4919727638	-0.5890899064	1.9710984106
5	C	0.3039688044	0.3931506693	1.3391288337
6	C	1.6069959204	0.2022189738	0.8230045111
7	C	2.5662155495	1.3224479503	0.6840193995
8	C	3.9331403544	0.6251286221	0.7315926571
9	C	3.6572407599	-0.7770715830	0.1577093188
10	C	2.1860657390	-0.9703450131	0.3194934214
11	C	1.5250156924	-2.1267882887	-0.1426721698
12	C	0.1737309760	-2.1842834441	-0.5603721366
13	S	-0.3366739136	-3.8556642139	-0.6890763749
14	C	1.2601534077	-4.4703091777	-0.3337114445
15	C	2.1208150461	-3.4188525803	-0.0902584076
16	Cl	-2.8231516735	2.8334829166	1.6158133958
17	C	-0.0951360203	-1.9204507443	2.4874317043
18	F	2.4641884316	2.2685634497	1.6364521787
19	F	2.4372140538	1.9818446937	-0.5016879892
20	F	4.3169979494	0.5193097371	2.0112952122
21	F	4.8817481215	1.2772245255	0.0622031472
22	F	4.4001428687	-1.7085907957	0.8066077478
23	F	4.0468980138	-0.8312745981	-1.1318566181
24	H	0.0642808153	2.5265016057	0.7830041877
25	H	-0.4052975164	-2.0319459723	3.5324932606
26	H	-0.5606510027	-2.7363614329	1.9145871317
27	H	0.9855082970	-2.0399136364	2.4244817394
28	C	-0.6660316901	-1.1064070489	-1.1474010259
29	H	-0.9934831696	-1.3842428261	-2.1580717658
30	H	-0.0821239845	-0.1878368022	-1.2204961986
31	H	-1.5673787714	-0.8891822395	-0.5600409584
32	C	1.5637561716	-5.8863692850	-0.2795567267
33	H	3.1586389182	-3.5576234696	0.1733942961
34	C	0.6076486007	-6.8556835318	-0.6155203426
35	C	2.8373234342	-6.3257447769	0.1182074667
36	C	3.1357525755	-7.6741539066	0.1767439827
37	C	2.1747857920	-8.6235231615	-0.1555628494
38	C	0.9099764310	-8.2047009981	-0.5508250910
39	H	-0.3815695605	-6.5507838706	-0.9368405652
40	H	3.5974386991	-5.6042190858	0.3880463076

41	H	4.1256346027	-7.9881649378	0.4871462828
42	H	0.1537907372	-8.9347638751	-0.8155129777
43	H	2.4106683039	-9.6801062243	-0.1075864248

CI'-S2/S1 Open-Form, MECP-S2/S1 optimized geometry. E_NN= 3623.16491482 hartrees

1	C	-1.6268725193	1.6188808682	1.6895597285
2	C	-0.3628394572	1.6611232027	1.2207251978
3	S	-2.0544589716	0.0500795390	2.3470244196
4	C	-0.4866152690	-0.5658589025	1.9909679362
5	C	0.3099322906	0.4028044750	1.3306944120
6	C	1.6102078486	0.1981183465	0.8061939688
7	C	2.5691306219	1.3167931795	0.6631519607
8	C	3.9358281393	0.6187651872	0.6853023393
9	C	3.6489943330	-0.7843486756	0.1189137383
10	C	2.1748523379	-0.9748488368	0.2934759849
11	C	1.5111119271	-2.1286126531	-0.1593386735
12	C	0.1462436158	-2.1932262701	-0.5322547021
13	S	-0.3560422555	-3.8746602857	-0.6225162342
14	C	1.2565101289	-4.4758183672	-0.3215573273
15	C	2.1169572816	-3.4221387796	-0.1180790946
16	Cl	-2.7761200225	2.8817881167	1.6678768394
17	C	-0.0976234370	-1.9025494125	2.4885584163
18	F	2.4818691588	2.2538532061	1.6273858427
19	F	2.4235352031	1.9924960919	-0.5119921221
20	F	4.3451055509	0.5155876669	1.9571233593
21	F	4.8711209530	1.2690626488	-0.0042834396
22	F	4.3908235309	-1.7147754354	0.7667506706
23	F	4.0231957152	-0.8444213246	-1.1732270357
24	H	0.0842797742	2.5320490323	0.7641434109
25	H	-0.4162018042	-2.0323709784	3.5286406606
26	H	-0.5648200505	-2.7065158458	1.8966802510
27	H	0.9821604695	-2.0284522916	2.4245366280
28	C	-0.7123112272	-1.1294594003	-1.1188067755
29	H	-1.0522665168	-1.4180614163	-2.1227892207
30	H	-0.1380613308	-0.2061055040	-1.2108319236
31	H	-1.6080929472	-0.9130230857	-0.5221172557
32	C	1.5696727284	-5.8938663519	-0.2673097388
33	H	3.1655384972	-3.5586435695	0.1021289816
34	C	0.6789657197	-6.8598613195	-0.7521003315
35	C	2.7840408179	-6.3303599331	0.2828246834
36	C	3.0920944839	-7.6775614788	0.3409211065
37	C	2.1957679298	-8.6262055142	-0.1403544075
38	C	0.9881638147	-8.2084983518	-0.6851110970
39	H	-0.2590563004	-6.5513999285	-1.1991962862
40	H	3.4849342172	-5.6066835187	0.6794840552
41	H	4.0359583795	-7.9911285889	0.7719469070
42	H	0.2833183667	-8.9377402024	-1.0679355228
43	H	2.4378282737	-9.6814053388	-0.0911903099

CI-S2/S1 Closed-Form, MECP-S2/S1 optimized geometry. E_NN= 3688.62690971 hartrees

1	C	-1.9813417796	0.6858205220	-0.4844705546
2	C	-0.6847666058	1.1046401988	-0.1616179646
3	S	-2.1582078584	-1.0236706520	-0.6681727982
4	C	-0.3949953763	-1.3122830912	-0.1803101631
5	C	0.2088326433	0.0675887271	-0.0605116156
6	C	1.6184578668	0.1188040699	0.0733594295
7	C	2.4569717751	1.2843052991	0.4123779436
8	C	3.7831830412	0.6336146950	0.8528659760
9	C	3.8362808491	-0.6825610602	0.0505882316
10	C	2.4027290204	-0.9913946730	-0.2128553760
11	C	1.8680253682	-2.1802326372	-0.7269141141
12	C	0.4496722704	-2.1064501818	-1.2434315066
13	S	-0.0535769131	-3.8643973369	-1.4459559647
14	C	1.5443876921	-4.4927909373	-1.0603548722
15	C	2.4137459022	-3.4508180806	-0.7028960447
16	Cl	-3.2911126706	1.7396944131	-0.7729438382
17	C	-0.3913083963	-2.0047575960	1.1848960921
18	F	1.9357428922	2.0686353519	1.3729440177
19	F	2.6880709076	2.0968972573	-0.6501208742
20	F	3.7139988371	0.3473428157	2.1604432273
21	F	4.8453041170	1.4063666701	0.6444055605
22	F	4.4778644571	-1.6543860399	0.7271611290
23	F	4.5213368625	-0.4883759640	-1.0925435605
24	H	-0.4157635300	2.1463178183	-0.0511213161
25	C	0.4091969271	-1.4174598532	-2.6093540168
26	C	1.8452949157	-5.8948668077	-1.0887702019
27	H	3.4241200637	-3.6264080199	-0.3613923444
28	C	0.8644155651	-6.8596235722	-1.3884220204
29	C	3.1502895287	-6.3535638176	-0.8126582259
30	C	3.4487401046	-7.7014062431	-0.8331477423
31	C	2.4644918379	-8.6410040873	-1.1273268997
32	C	1.1720060245	-8.2069236428	-1.4046554883
33	H	-0.1496949097	-6.5475443201	-1.6097431474
34	H	3.9351664438	-5.6440609018	-0.5860882094
35	H	4.4608189829	-8.0250759927	-0.6183496604
36	H	0.3960956616	-8.9274244361	-1.6370493813
37	H	2.7028408879	-9.6978520408	-1.1419381031
38	H	-0.9178260349	-1.3870943557	1.9150279498
39	H	0.6406621511	-2.1445210443	1.5150280764
40	H	-0.8768262614	-2.9805742800	1.1163471518
41	H	0.7544877039	-0.3847973683	-2.5093275991
42	H	-0.6063288808	-1.4136124154	-3.0120696879
43	H	1.0674979161	-1.9440263891	-3.3024314938

CI-S1/S0 from the highest energy barrier of PES, MECP-S1/S0 optimized geometry. E_NN= 3689.66011685 hartrees

1	C	-1.9146592044	0.7260861093	-0.5481092819

2	C	-0.7098776327	1.1110443644	-0.1344827404
3	S	-2.1184349125	-1.0481755335	-0.5206021720
4	C	-0.4568476944	-1.3009356179	0.0143387173
5	C	0.1808332711	0.0116339841	0.1658041888
6	C	1.5458862573	0.0729147963	0.1119307423
7	C	2.4042653793	1.2676578701	0.3780563332
8	C	3.7218847303	0.6336073432	0.8621799513
9	C	3.7934565213	-0.7166716319	0.1176125322
10	C	2.4038532083	-0.9846479955	-0.2372629259
11	C	1.8837173520	-2.1649652874	-0.8289195464
12	C	0.6393992911	-2.1319604735	-1.5430985737
13	S	0.2006343122	-3.8007913853	-1.9588608165
14	C	1.5973044277	-4.4371902394	-1.2034211863
15	C	2.4028264478	-3.4538979480	-0.6693682659
16	Cl	-3.2375066067	1.7032430910	-1.0566170379
17	C	-0.2790918713	-2.3441847365	1.0891294017
18	F	1.9016381647	2.1352145541	1.2644687638
19	F	2.6252801260	1.9490122995	-0.7665873734
20	F	3.6250657073	0.4172607510	2.1831030539
21	F	4.7791865156	1.4124563640	0.6418625762
22	F	4.3767593022	-1.6712919138	0.9057080568
23	F	4.6380397509	-0.6105355565	-0.9445064766
24	H	-0.3886846787	2.1434829155	-0.0955554379
25	C	0.4257531421	-1.1307493896	-2.6537159617
26	C	1.8521747059	-5.8730693652	-1.1192412656
27	H	3.3006173112	-3.6540816997	-0.1032667049
28	C	0.8157507381	-6.8063347883	-1.2078063613
29	C	3.1639544423	-6.3269616763	-0.9512641795
30	C	3.4314832040	-7.6831828389	-0.8812165523
31	C	2.3937248884	-8.6029769099	-0.9672724217
32	C	1.0861907472	-8.1616504057	-1.1288811771
33	H	-0.2076955167	-6.4676537628	-1.3183928085
34	H	3.9761192973	-5.6132369557	-0.8981043805
35	H	4.4529563195	-8.0235405924	-0.7610339247
36	H	0.2740257553	-8.8760885541	-1.1898506796
37	H	2.6039344416	-9.6645540627	-0.9079670759
38	H	-0.9068325257	-2.0563586033	1.9367314288
39	H	0.7588583381	-2.3643902498	1.4214200511
40	H	-0.5769103795	-3.3392375964	0.7524827193
41	H	0.6866403811	-0.1342817663	-2.2973301813
42	H	-0.6224795523	-1.1258832534	-2.9661359910
43	H	1.0557861978	-1.3640636531	-3.5174566165

TS-S0, Transition state geometry. E_NN= 3689.69084292 hartrees

1	C	-1.8400804057	0.8782176088	1.2929471735
2	C	-0.5196814304	1.2300268583	1.2311589234
3	S	-2.0856714544	-0.8336132434	1.4627479897
4	C	-0.3222502994	-1.1604135000	1.3628522006
5	C	0.3371897746	0.1307159576	1.2998818064

6	C	1.7338068535	0.1688772026	0.9329057964
7	C	2.6412989797	1.3313493667	1.1247781265
8	C	4.0361563006	0.7535262540	0.8190482828
9	C	3.7460218405	-0.4536274348	-0.0939914908
10	C	2.3360819616	-0.8051828860	0.2213818019
11	C	1.6396277624	-2.0093397050	-0.1975161374
12	C	0.2008384686	-1.9273444918	-0.3704987601
13	S	-0.3757900701	-3.6042764146	-0.5726800260
14	C	1.1963347561	-4.2972484212	-0.2704738519
15	C	2.1417191802	-3.2928249920	-0.1186816651
16	Cl	-3.1712242941	1.9464371844	1.1378929665
17	C	0.1436366333	-2.1836557868	2.3744965812
18	F	2.5786134520	1.8632822275	2.3525995301
19	F	2.3486590671	2.3267792423	0.2556357168
20	F	4.5880486709	0.3232758693	1.9600865200
21	F	4.8539990691	1.6402248984	0.2586998901
22	F	4.6062958006	-1.4656815516	0.1398877617
23	F	3.9012189120	-0.1067926133	-1.3850572788
24	H	-0.1800326525	2.2467205964	1.0845971189
25	H	-0.0535893787	-1.8165468255	3.3845855632
26	H	-0.3729952280	-3.1362194702	2.2352659958
27	H	1.2160131187	-2.3511994683	2.2688535563
28	C	-0.3744376781	-0.9625395912	-1.3821349987
29	H	-0.0943269503	-1.2768405768	-2.3905356400
30	H	0.0236914260	0.0379944424	-1.2082876924
31	H	-1.4639605596	-0.9231247741	-1.3123593137
32	C	1.4129909440	-5.7276981020	-0.1533106732
33	H	3.1793075789	-3.4912250156	0.1104575137
34	C	0.3459414918	-6.6298429747	-0.0603101544
35	C	2.7171775821	-6.2422278440	-0.1289849474
36	C	2.9410494118	-7.6018651396	-0.0107286034
37	C	1.8718947108	-8.4856039935	0.0844904932
38	C	0.5749154863	-7.9907520989	0.0577931796
39	H	-0.6738906752	-6.2627745252	-0.0692322126
40	H	3.5619629764	-5.5707932853	-0.2155529472
41	H	3.9584396869	-7.9757480396	0.0028227196
42	H	-0.2674835883	-8.6689032916	0.1316624455
43	H	2.0492426684	-9.5507215520	0.1768266399

Benchmarking between SF-TDDFT(BHHLYP), ADC(2) and XMS-CASPT2

Table 1 summarizes the results obtained for the two lowest singlet excited states of the cyclohexadiene (CHD) chromophore using SF-TDDFT(BHHLYP), ADC(2) and XMS-CASPT2 methods. We can notice that SF-BHHLYP shows a reasonable performance compared with the XMS-CASPT2 method and in particular the experimental results. Furthermore, comparing SF-BHHLYP with ADC(2), SF-BHHLYP shows results closer to the experimental ones than ADC(2), in particular for S_2. ADC(2) shows a reasonable performance compared with the XMS-CASPT2 method, in particular for S_1.

Table 1. Vertical excitation energies in eV for the two lowest singlet excited states of the cyclohexadiene (CHD) chromophore.

Method	CHD		Ref.
	S1(1B)	S2(2A)	
SF-BHHLYP	5.26	5.89	[1]
XMS-CASPT2	5.13	6.28	[2]
ADC(2)	5.44	7.26	
Experiment	4.94	6.30	[3]

Non-symmetric DTE and symmetric counterparts

Photoisomerization quantum yields (QYs) and absorption maximum wavelengths for the non-symmetric DTE and its symmetric counterparts are describe in Table 2.

Table 2. Absorption maximum wavelengths (λ_{max}) and quantum yields of the photocyclization ($\Phi_{O\to C}$) and photoreversion ($\Phi_{C\to O}$) processes of a non-symmetric DTE bearing phenyl/chlorine substituent groups and its symmetric counterparts. This Table is base on Table 1 in Ref.[4].

**DTE**	**Open form** λ_{max}**(nm/eV)**	**Closed form** λ_{max}**(nm/eV)**	$\Phi_{O\to C}$	$\Phi_{C\to O}$	Refs.
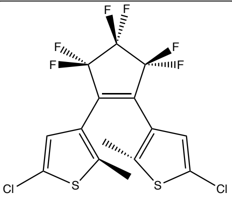	242/5.12	504/2.42	0.47	0.13	[5, 6]
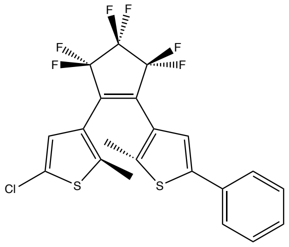	255/4.86	548/2.26	—	—	[5]
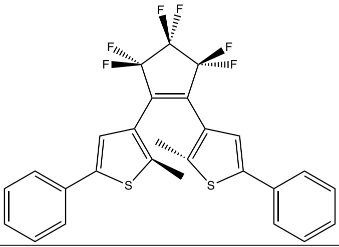	280/4.43	575/2.16	0.59	0.013	[4, 5, 7]

Natural Transiton Orbitals in solvent

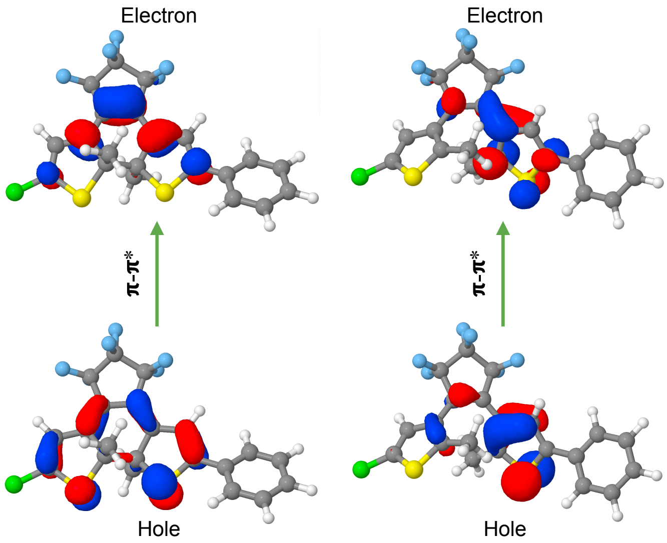

(a) DTE closed-ring S_1 (b) DTE open-ring S_2

Figure 1. NTOs calculated with SF-TDDFT(BHHLYP)/cc-pVDZ (cutoff value of 0.04) in solvent (acetonitrile) for the lowest bright state with significant oscillator strength.

Analysis of energies and derivative couplings

Potential energies curves, norm of derivative coupling and distance between the reaction carbons C_1-C_2 along the MECPs optimisation trajectories are plotted in Figures 2-3.

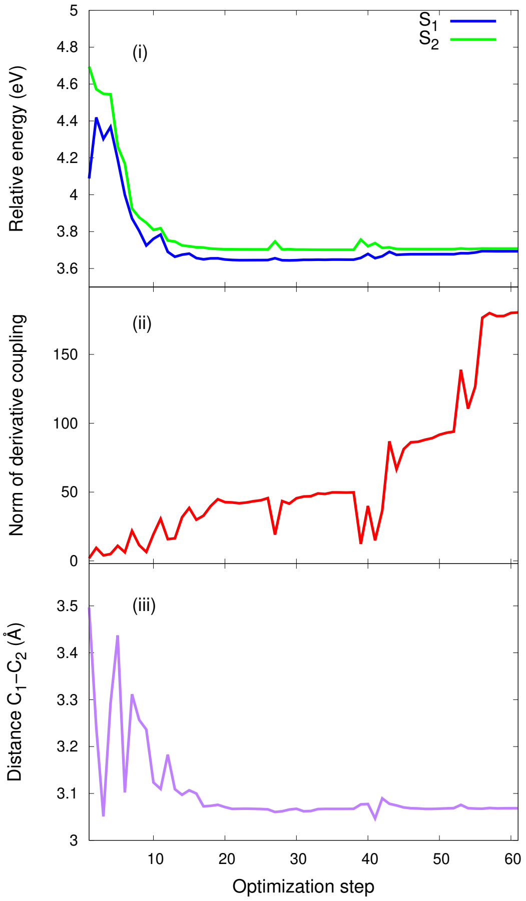

(a) $CI_{S2/S1}$ Open form

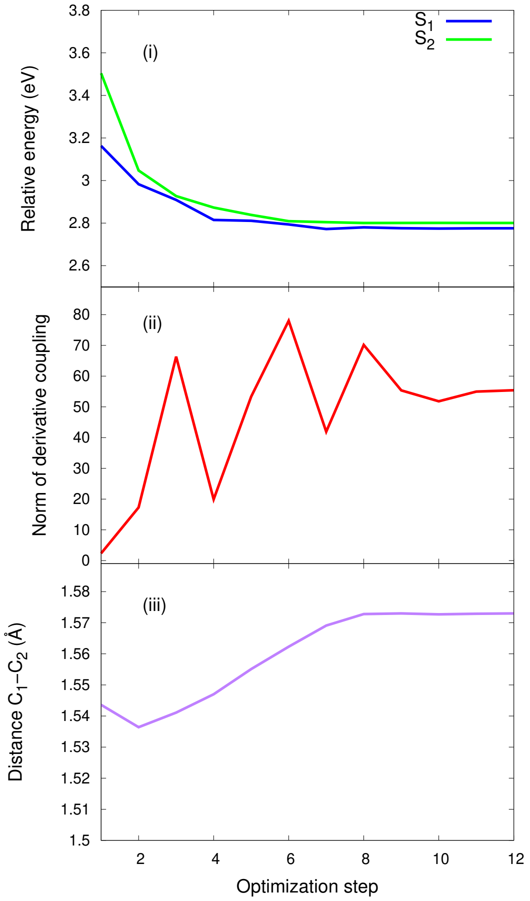

(b) $CI_{S2/S1}$ Closed form

Figure 2. (a) $CI_{S2/S1}$ Open form: potential energies curves (i), norm of derivative coupling (ii) and distance between the reaction carbons C_1-C_2 (iii) along the S_2/S_1 MECP optimization trajectory of the DTE open form. (b) $CI_{S2/S1}$ Closed form: potential energies curves (i), norm of derivative coupling (ii) and distance between the reaction carbons C_1-C_2 (iii) along the S_2/S_1 MECP optimization trajectory of the DTE closed form. The energies are relatives to the S_{0min} energy of the DTE open form.

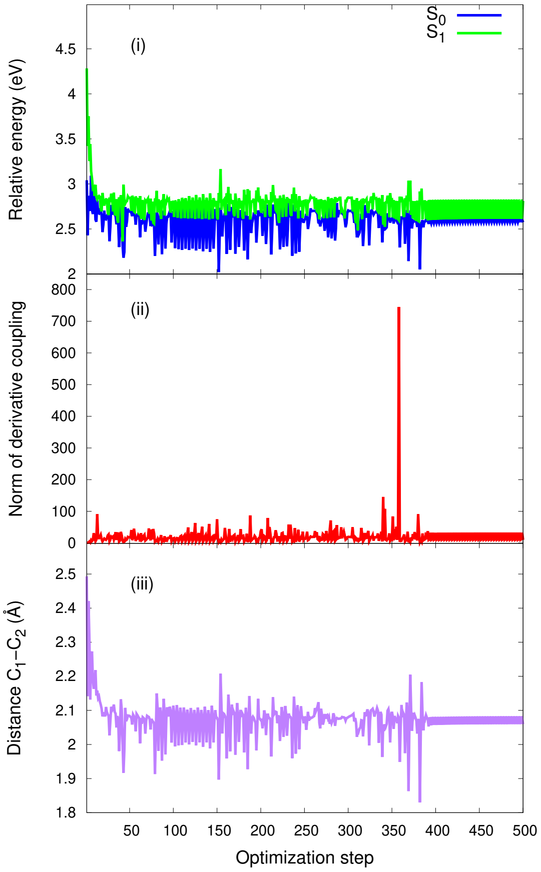

Figure 3. $CI_{S1/S0}$: potential energies curves (i), norm of derivative coupling (ii) and distance between the reaction carbons C_1-C_2 (iii) along the S_1/S_0 MECP optimization trajectory from the geometry associated to the highest barrier energy obtained on the relaxed PES scan on S_1 state. The energies are relatives to the S_{0min} energy of the DTE open form.

Potential Energy Surface scans

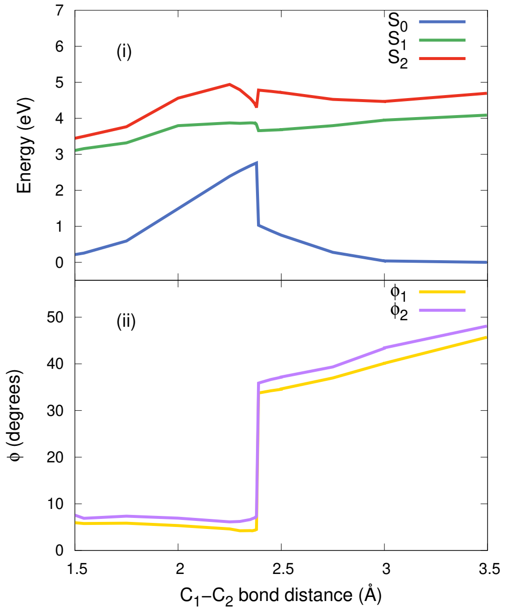

(a) Relaxed S_0-PES scan

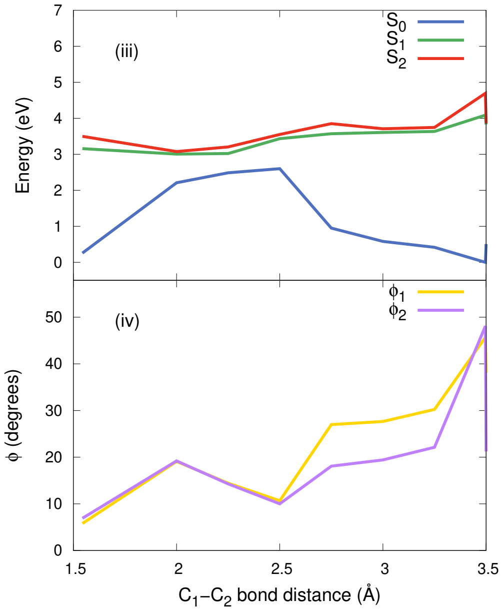

(b) Relaxed S_2-PES scan

Figure 4. (a) Relaxed S_0-PES scan: potential energies curves (i) and ϕ torsion angles (ii) along the C_1-C_2 bond distance obtained with SF-BHHLYP/cc-pVDZ. (b) Relaxed S_2-PES scan: potential energies curves (iii) and ϕ torsion angles (iv) along the C_1-C_2 bond distance obtained with SF-BHHLYP/cc-pVDZ. The energies are relatives to the S_{0min} energy of the open-ring.

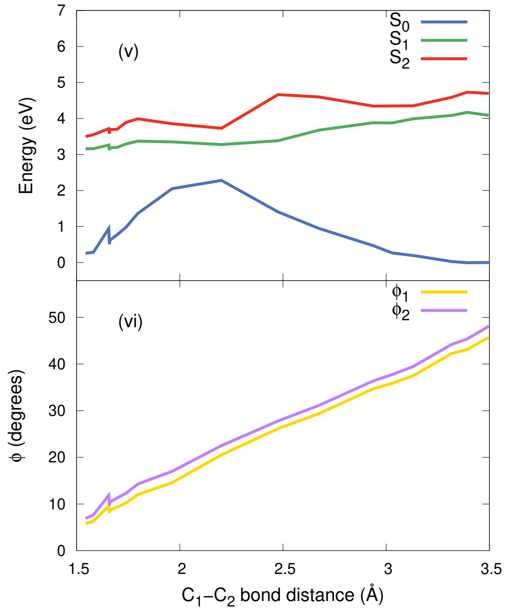

Figure 5. Rigid-PES scan: potential energies curves (v) and ϕ torsion angles (vi) along the C_1-C_2 bond distance obtained with SF-BHHLYP/cc-pVDZ. The energies are relatives to the S_{0min} energy of the open-ring.

References

[1] E. Salazar and S. Faraji, *Mol. Phys.*, 2020, **118**, e1764120.

[2] I. Polyak, L. Hutton, R. Crespo-Otero, M. Barbatti and P. J. Knowles, *J. Chem. Theory Comput.*, 2019, **15**, 3929–3940.

[3] M. Merchán, L. Serrano-Andrés, L. S. Slater, B. O. Roos, R. McDiarmid and Xing, *J. Phys. Chem. A*, 1999, **103**, 5468–5476.

[4] M. Irie, T. Fukaminato, K. Matsuda and S. Kobatake, *Chem. Rev.*, 2014, **114**, 12174–12277.

[5] W. R. Browne, J. J. D. de Jong, T. Kudernac, M. Walko, L. N. Lucas, K. Uchida, J. H. van Esch and B. L. Feringa, *Chem. Eur. J.*, 2005, **11**, 6430–6441.

[6] K. Higashiguchi, K. Matsuda, Y. Asano, A. Murakami, S. Nakamura and M. Irie, *Eur. J. Org. Chem.*, 2005, **2005**, 91–97.

[7] M. Irie, T. Lifka, S. Kobatake and N. Kato, *J. Am. Chem. Soc*, 2000, **122**, 4871–4876.
